# Implementation of an integrated home internet of things system for vulnerable older adults using a frailty-centered approach

**DOI:** 10.1038/s41598-022-05963-9

**Published:** 2022-02-04

**Authors:** Ji Yeon Baek, Se Hee Na, Heayon Lee, Hee-Won Jung, Eunju Lee, Min-Woo Jo, Yu Rang Park, Il-Young Jang

**Affiliations:** 1 Division of Geriatrics, Department of Internal Medicine, Asan Medical Center, University of Ulsan College of Medicine, 88 Olympic-ro 43-gil, Songpa-gu, Seoul, 05505 Republic of Korea; 2 Department of Biomedical System Informatics, Yonsei University College of Medicine, 50-1 Yonsei-ro Seodaemun-gu, Seoul, 03722 Republic of Korea; 3 Division of Pulmonary, Critical Care and Sleep Medicine, Department of Internal Medicine, Eunpyeong St. Mary’s Hospital, The Catholic University of College of Medicine, Seoul, Republic of Korea; 4 Department of Preventive Medicine, University of Ulsan College of Medicine, Seoul, Republic of Korea

**Keywords:** Geriatrics, Health services

## Abstract

Although integrated home internet of things (IoT) services can be beneficial, especially for vulnerable older adults, the hurdle of usability hinders implementation of the technology. This study aimed to evaluate the practical usability of home IoT services in older adults, by frailty status, and to determine the potential obstacles. From August 2019 to July 2020, we randomly selected 20 vulnerable older adults (prefrailty group [*n* = 11], and frailty group [*n* = 9]) who had already been identified as needing home IoT services in a community-based prospective cohort study, the Aging Study of the Pyeongchang Rural Area. Integrated home IoT services were provided for 1 year, and a face-to-face survey evaluating usability and satisfaction of each service was conducted. The usability of the integrated home IoT services declined gradually throughout the study. However, prefrail participants showed higher usability than frail older adults (difference-in-difference = − 19.431, *p* = 0.012). According to the frailty status, the change in usability for each service type also showed a different pattern. During the 12-month study period, the service with the highest satisfaction converged from various service needs to light control by remote control (77.8%) in the prefrailty group and automatic gas circuit breaker (72.7%) in the frailty group. For wider implementation of home IoT services, organizing services expected to have high usability and satisfaction based on user’s frailty status is crucial. Also, providing education before service implementation might help older adults coping with digital literacy.

## Introduction

Population aging is a global phenomenon that is increasing life expectancy. This overwhelming global aging has consequently resulted in an increase in the number of frail older adults. Frail older adults show marked declines in both physical and psychological function, and have a high proportion of disability and high incidence of disease-related complications compared with other older adults. Hence, population aging increases care burden: nursing home use, institutionalization, and hospitalization, which eventually leads to high social costs.

According to several national studies, most older adults would prefer to age in place, which means living in their own homes with autonomy and independence^1–3^. For frail older adults, aging in place requires special assistance and services because of their lack of independence. However, limited resources make it hard to fulfill the various needs of older adults even with the cooperative help of community and society. Therefore, the role of technology-incorporated healthcare services and devices is increasingly growing in the field of older adult care^4^. Though many studies have shown favorable effects of such services and devices in community-dwelling older adults^5–8^, there are still numerous hurdles, such as unawareness of the devices, complexity of systems, invasion of privacy, and low adherence, hindering wider implementation of technology-incorporated devices and services such as home internet of things (IoT)^9,10^.

Unlike younger people, older adults have diverse health status^11,12^. Neither chronological age or comorbidities can provide a complete picture of physical and functional status in older adults, and there exists the need for a specific marker of overall health status in this population. Among the many markers, frailty is regarded as the most representative, involving physical and cognitive function, and comorbidities burden; it can describe physiologic reserve and quantify vulnerabilities to stressors^13^. Frailty has already been well validated to predict not only mortality but also adverse health outcomes, falls, hospitalization, and even quality of life in diverse clinical settings, including communities, hospitals, and nursing homes^14–17^. Recently, the frailty-based approach is used by the British National Health Service for making clinical decisions about treatment modality and intensity for older adults with severe diseases^18^. Of note, the frailty-based approach has shown promising results when used in wearable device-based walking programs, maintaining high usability and retention rates especially in older adults^19^.

Given the fact that older adults living in the community are unable to fully benefit from home IoT services despite having the highest necessity, the frailty-based approach may be necessary in the field of implementation of Home IoT services. Therefore, as a feasibility study, we observed longitudinal changes of usability and satisfaction levels of home IoT services in vulnerable older adults who would benefit most from using home IoT services by the frailty status. The primary outcome was the usability and satisfaction level of home IoT services itself in vulnerable older adults by their frailty status, and the secondary outcomes were changes in health-related benefits and care burden.

## Methods

### Study design and population

As a feasibility study, participants were recruited from the Aging Study of the Pyeongchang Rural Area, a community-based prospective cohort study of Korean older adults from August 2019 to July 2020, and detailed description of the cohort has been provided elsewhere^20^.

We have previously surveyed home IoT service needs using the same cohort^21^. Among these participants, we randomly selected 20 vulnerable subjects who were expected to benefit most from using home IoT service with the highest need based on our previous study^21^, given practical concern about limited resources. Individuals were included if they (1) frail or prefrail older adults according to the Cardiovascular Health Study (CHS) frailty phenotype scale; (2) living alone or spending more than eight hours alone during the day; (3) a smart phone user using less than 20% of the phone’s capabilities or not a smart phone user; (4) did not have installed communication infrastructure at home such as wireless fidelity and optical networking. Subjects who were unable to communicate verbally, or who were in robust health, as determined with the CHS frailty scale, were excluded.

In this study, integrated home IoT services were provided for 12 months, and a face-to-face survey was conducted. Data for the survey were collected at baseline, and at 1, 6, and 12 months. All participants provided written informed consent. This study was approved by the institutional review board of the Asan Medical Center, Seoul, Korea (IRB No. 2019-0041) and was conducted in accordance with the Helsinki Declaration of 1975, as revised in 2000.

### Assessment of frailty

Frailty was assessed by the CHS frailty scale which is widely used in current frailty research for its validity and reliability^13,14,22^. This frailty scale consists of the five items: (1) losing weight more than 4.5 kg unintentionally last year, (2) decrease in hand grip strength, being in the lowest 20% in consideration of sex and body mass index^23^, (3) feeling exhausted on the Korean version of Center for Epidemiological Studies Depression Scale (CES-D)^24^, (4) slowness in walking, being in the lowest 20% in consideration of sex and height^23^, (5) decline in physical activity evaluated by an International Physical Activity Questionnaire score: below 494.65 kcal/week for males and 283.5 kcal/week for females^23^. Having more than three of the above items was defined as frailty and having on or two items was defined as prefrailty.

### Development of the integrated home IoT system

**Figure 1:**
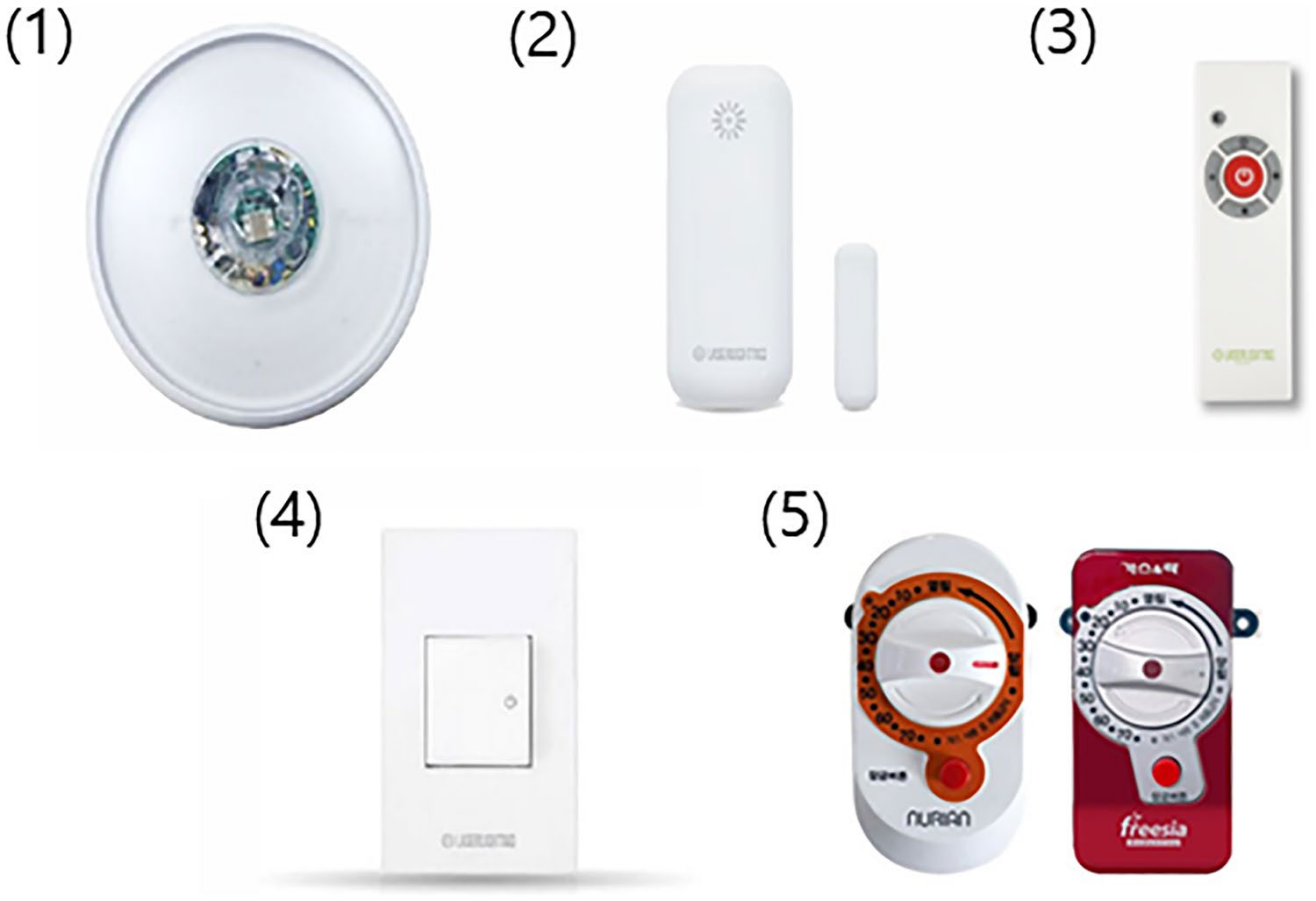
Five Internet of Things (IoT) devices used in the study. (1) Human sensing LED lighting (entrance ceiling) senses user’s motion and turns on the light, (2) Occupancy sensor (door) also detects user’s movement, (3) Remote controller has buttons for turning on/off the lights and making SOS call, (4) Wall switch can turn on/off the light and send usage log to communication module in LED lighting, (5) Automatic gas circuit breaker blocks a gas flow when an individually set time passes. Data (usage log of LED lighting, measurement value of occupancy sensor) from all devices except the automatic gas circuit breaker, are all gathered in communication module of LED lighting, and sent to a main server every 5 min without a personal home wired or wireless network. *LED* light emitting diode.

Integrated home IoT system services were provided for 12 months to 20 participants. Based on the results of our previous research assessing the demand for home IoT in vulnerable older adults^21^, we selected previously commercialized services or devices that were affordable and easy to operate for public health centers and developed an integrated home IoT system. This system consisted of five devices with eight specific functions (Fig. 1). The five devices were as follows: (1) human-sensing light-emitting diode (LED) on the entrance ceiling, (2) occupancy sensor on the door, (3) light control and emergency SOS call by remote control, (4) wall switch, and (5) automatic gas circuit breaker. The eight specific functions were as follows: (1) motion sensing by the sensor on the entrance ceiling lighting, (2) light control by remote control, (3) SOS call by remote control, (4) occupancy sensing by the sensor on the door, (5) sending data from the wall switch sensor, (6) emergency alarm service via text message when there were no detected movements from the participant for more than 12 h, (7) text-to-speech (TTS) service from the service manager to participants at the emergency situation, and (8) automatic gas circuit breaking when an individually set time passes.

For the LED lighting, a communication module was embedded that enabled the device to be operated without a personal home wired or wireless network. This module gathered all the data from the devices except the automatic gas circuit breaker, such as the LED lighting usage log from the remote control and wall switch, and measurement values of the occupancy sensor, and sent them to a main server every 5 min. Then, the data were integrated to determine whether the home was occupied by the participant. If activity was not detected for more than 12 h, an alert text message was sent to a designated family member or a matched care provider at a public health center. Tracking log and alertness activation history could be monitored through a main server at the public health center. All the services could operate automatically without a smart phone, and there were no additional operating requirements except for a power supply.

### Outcome assessment

#### Questionnaires

Trained social workers administered a face-to-face survey with a questionnaire at baseline, and at 1, 6, and 12 months of the study. The questionnaire included 20 questions related to system usability, willingness to pay for the service, health-related benefits, care burden, and the most satisfying service. For measuring service usability, a validated and simple 10-items usability scale, the System Usability Scale (SUS) was used^25,26^. The SUS assesses various aspects of system usability, including effectiveness, efficiency, and satisfaction. The total score ranges from 0 (“low usability”) to 100 (“high usability”); and a score over 70 is related to “good” in adjective ratings and “acceptable” in acceptability ranges^26^.

#### Comprehensive geriatric assessment

For measuring physical performance, the Short Physical Performance Battery (SPPB) comprising five times sit-to-stand test, standing balance test, and gait speed, was used^27^. Each section of the SPPB is scored out 4 points, with a total of 0 (“worst”) to 12 (“best”). In accordance with previous reports, lower SPPB score was defined as lower than 10^28^. Grip strength was measured using a hand-held dynamometer (TKK 5401 Grip-D; Takei, Tokyo, Japan) in a seated position with the elbow flexed at 90°. The measurement was conducted twice, and the maximal power of the dominant arm was recorded.

Disability was evaluated by the Korean versions of the activities of daily living (ADL) and instrumental activities of daily living (IADL) scales which were both validated for Korean population. The ADL scale consists of seven fundamental items for living independently, such as eating, bathing, toileting, dressing, continence, washing face and hands, and ambulation. The IADL scale consists of ten items, such as making phone calls, shopping, preparing meals, handling finances, household chores, going out a short distance, using public transportation, taking medications, and grooming^29^. Limitation in ADL and/or IADL was defined as needing other people’s help for doing any of the above tasks^29^. Nutritional status was assessed by the Mini-Nutritional Assessment Short Form, and a score lowering than 11 points was regarded as having a risk of malnutrition^30^. Cognitive function was evaluated by the Korean version of the Mini-Mental State Exam, with a score ranging from 0 to 30. A Mini-Mental State Exam score under 24 was defined as cognitive dysfunction^31^. For determining depression, the Korean version of the CES-D was used. The CES-D scores from 1 to 60, and scoring more than 21 points was defined as depression^32^.

### Statistical analysis

Initial data analysis compared the differences in geriatric parameters according to frailty status. Because the total number of participants did not exceed 30, we performed a nonparametric Mann–Whitney–Wilcoxon test and Fisher test for mean and ratio evaluation, respectively. A difference-in-difference (DID) analysis was carried out to determine the effect of the IoT devices in terms of usability and satisfaction level according to frailty status and time^33^. Therefore, we conducted a DID analysis between the SUS of the baseline survey and the SUS of the survey conducted at 1, 6, and 12 months. The willingness to pay for each service at the specific timepoints was also comparatively analyzed according to frailty status using the Mann–Whitney–Wilcoxon test. Data analyses were conducted using R software, version 4.1.0 (R Foundation, Vienna, Austria). We regarded two-sided *p* values < 0.05 as statistically significant.

### Ethics approval and consent to participate

This study was approved by the institutional review board of the Asan Medical Center, Seoul, Korea. Informed consent was obtained from all study participants.

## Results

### Overall characteristics of the population

This study included a total of 20 participants with a mean age of 79.95 years. Ninety percent were female. Participants with prefrailty and frailty were 55% (*n* = 11) and 45% (*n* = 9), respectively. Most of the participants had no formal education (70%) and lived alone (65%). Mean body mass index was 25.12 kg/m^2^. Multimorbidity existed in 90% of the participants, and hypertension and arthritis were the two most common diseases (Table 1).

**Table 1:** Basic characteristics of the study participants.

Characteristic	n (%) or mean (SD)
Number	20
Age (years)	79.95 (5.28)
Female	18 (90%)
Body mass index (kg/m^2^)	25.12 (3.14)
**Currently working**	6 (30%)
Engaged in agriculture	5 (25%)
Service/office job	1 (5%)
**Education level**	
No formal education	14 (70%)
≤ 6 years (elementary school)	5 (25%)
7–12 years (middle or high school)	0 (0%)
> 12 years (college or higher)	1 (5%)
**Living status**	
Living with spouse	7 (35%)
Living alone	13 (65%)
**Frailty status by CHS criteria**	
Prefrail	11 (55%)
Frail	9 (45%)
**Multimorbidity**	18 (90%)
Hypertension	19 (95%)
Diabetes	7 (35%)
COPD	1 (5%)
Myocardial infarction	1 (5%)
Heart failure	1 (5%)
Angina	2 (10%)
Asthma	3 (15%)
Arthritis	16 (80%)
Stroke	3 (15%)
Chronic kidney disease	1 (5%)

*CHS* cardiovascular health study, *COPD* chronic obstructive pulmonary disease, *SD* standard deviation.

Results of the geriatric assessment indicated differences between the prefrailty and frailty groups in terms of physical function and depression (Table 2). Mean SPPB score was lower in the frailty group compared with the prefrailty group (*p* < 0.016). Gait speed and grip strength were also significantly lower in the frailty group compared with the prefrailty group (*p* = 0.002, 0.001, respectively). There were no significant differences in ADL disability, IADL disability, or other geriatric conditions, such as malnutrition and cognition. However, depressive mood, measured with the CES-D, was higher in the frailty group compared with the prefrailty group (*p* = 0.006).

**Table 2:** Geriatric parameters by frailty status.

	Prefrailty group (n = 11)	Frailty group (n = 9)	Total (n = 20)	*p* value
Short Physical Performance Battery score	8.09 (3.02)	5.56 (2.79)	6.95 (3.12)	0.016
Gait speed (m/s)	0.84 (0.26)	0.49 (0.17)	0.69 (0.28)	0.002
Grip strength (kg)	18.69 (4.22)	12.30 (2.13)	15.82 (4.68)	0.001
ADL disability	7 (63.6%)	7 (77.8%)	14 (70.0%)	0.844
IADL disability	3 (27.3%)	6 (66.7%)	9 (45.0%)	0.190
At risk of malnutrition (MNA-SF)	3 (27.3%)	1 (11.1%)	4 (20.0%)	0.606
Cognition (MMSE score)	24.09 (4.46)	22.88 (2.37)	23.55 (3.63)	0.271
Depression (CES-D score)	6.46 (15.24)	18.67 (12.01)	11.95 (14.89)	0.006

Values are presented as mean ± standard deviation or number (%).

*MMSE* Mini-Mental State Examination, *CES-D* Center for Epidemiological Studies Depression Scale, *MNA-SF* Mini-Nutritional Assessment-Short Form, *ADL* activities of daily living, *IADL* instrumental activities of daily living.

### Usability of the services

**Figure 2:**
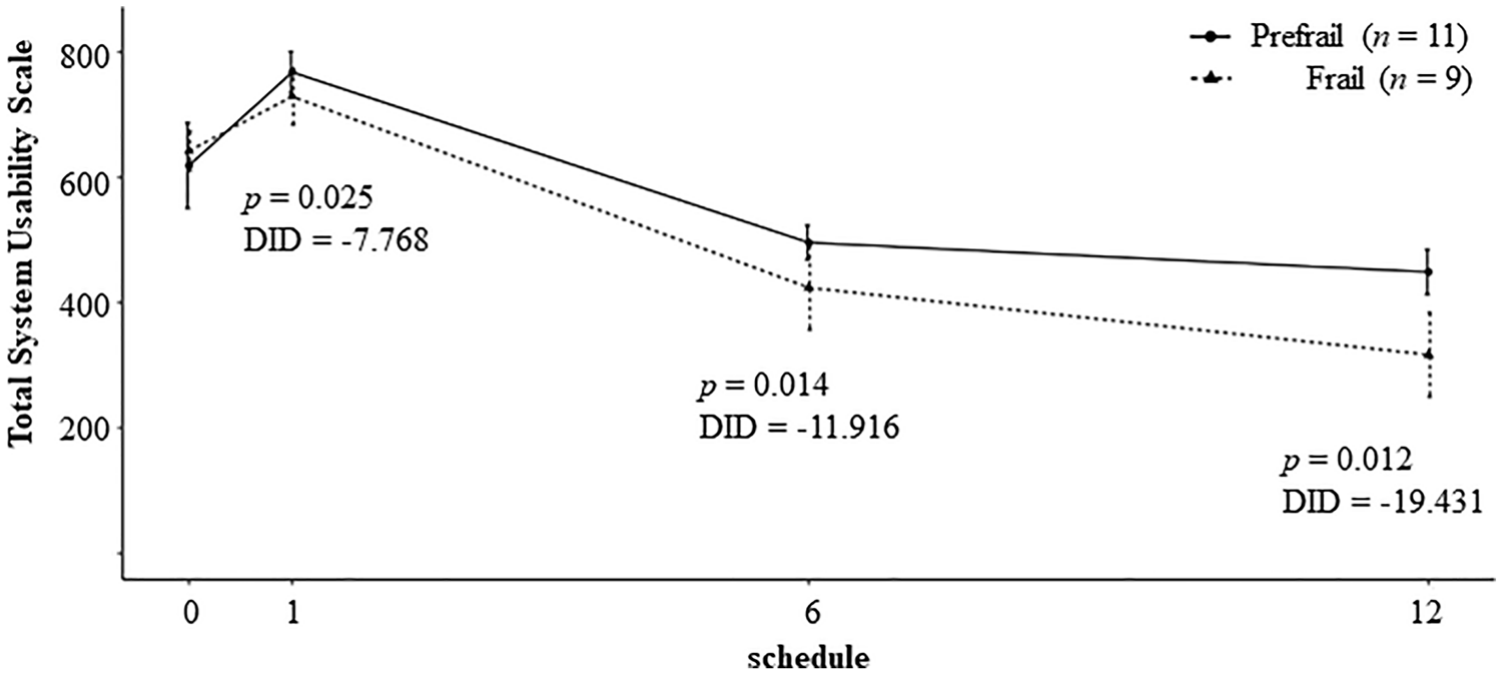
Total system usability scale by frailty status. The usability of the whole services gradually decreased throughout the study period; however it was relatively higher in prefrail group compared with frail group. DID, difference-in-difference. Data preprocessing was conducted by using Python software, version 3.6.8 (The Python Software Foundation, DE, USA), URL https://www.python.org, and the figure was created by using R software, version 4.1.0 (R Foundation, Vienna, Austria), URL https://www.r-project.org.

Changes in SUS score throughout the study are presented in Fig. 2. In both groups, the mean SUS score increased, peaking at 1 month, and gradually decreased throughout the remainder of the study. However, mean SUS was relatively lower in the frailty group compared with the prefrailty group over time, and a gap of DID estimation between the prefrailty and frailty group became wider throughout the study. DID discrepancy was − 7.768 (*p* = 0.025) at 1 month, − 11.916 (*p* = 0.014) at 6 months, and − 19.431 (*p* = 0.012) at 12 months. The final SUS score at 12 months concluded as “good” in relation with adjective ratings and “acceptable” in acceptability ranges only in the prefrailty group, not in the frailty group (the mean SUS score > 70)^26^.

**Figure 3:**
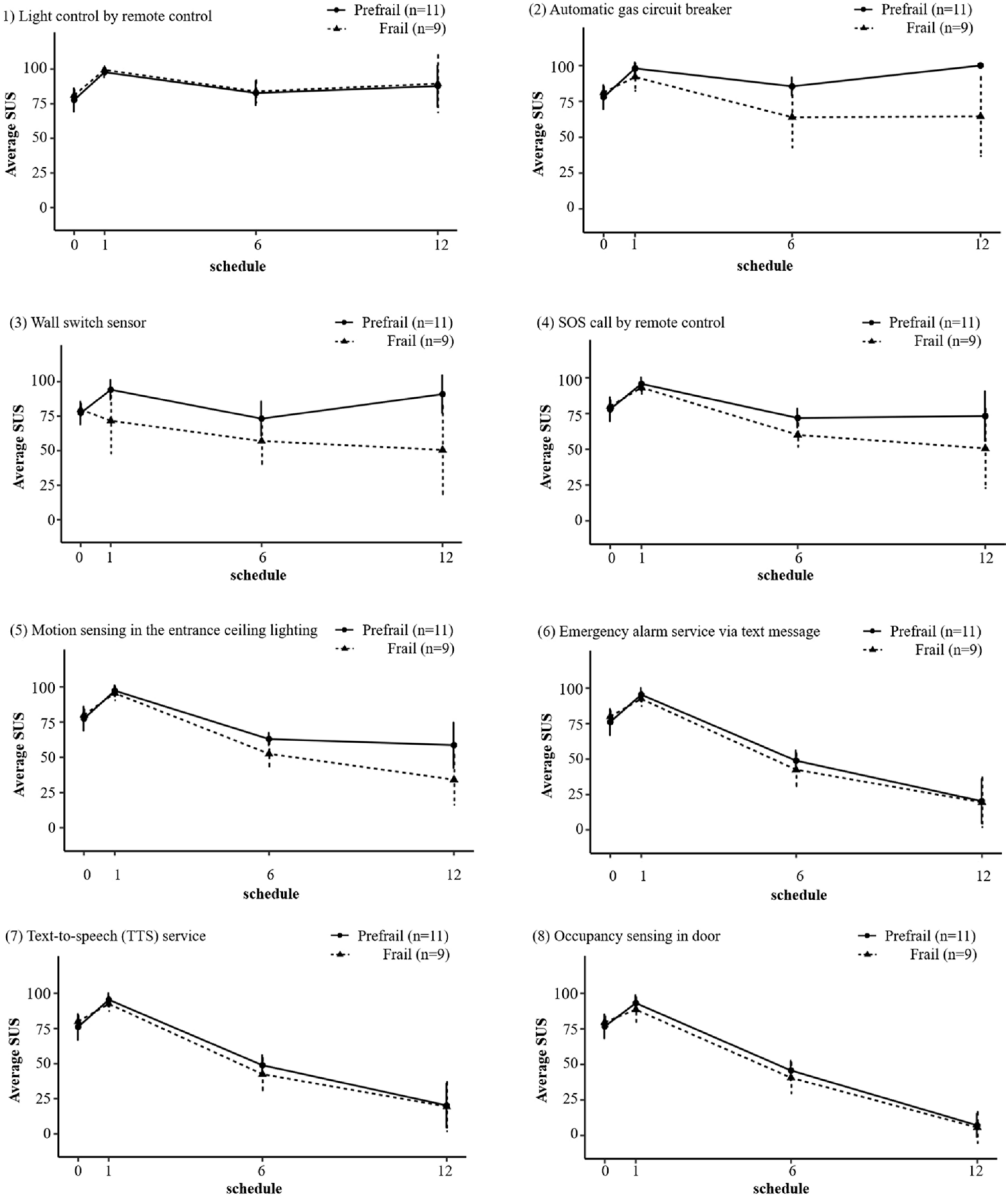
Service-specific system usability scale by frailty status. The usability pattern was different according to the type of services. Light control by remote controller showed consistently high usability both in the prefrail and frail group. Certain services such as automatic circuit breaking, light control by wall switch sensor, SOS call by remote controller, and motion sensing by the sensor in the entrance ceiling lighting showed a down slope in both groups, but relatively higher in prefrail group. Usability of the emergency text alarm service, text-to-speech service, and occupancy sensing by the door sensor decreased steadily both in the prefrail and frail group. *SUS* System Usability Scale. Data preprocessing was conducted by using Python software, version 3.6.8 (The Python Software Foundation, DE, USA), URL https://www.python.org, and the figure was created by using R software, version 4.1.0 (R Foundation, Vienna, Austria), URL https://www.r-project.org.

Changes in service-specific usability in both groups are presented in Fig. 3. For most services, the mean SUS score was higher in the prefrailty group compared with the frailty group. However, changes in SUS score differed between services over time. Remote light control showed the highest usability continuously throughout the entire study in both the prefrailty and frailty groups. However, the usability of certain services (automatic gas circuit breaking, light control by wall switch sensor, SOS call by remote control, and motion sensing by the sensor in the entrance ceiling lighting) showed a down slope in both groups, and adjectively rated as “good” and “acceptable in the prefrailty group (the mean SUS score > 70)^26^. The gaps between the prefrailty and frailty groups widened throughout the study. Lastly, usability of the emergency text alarm service, TTS service, and occupancy sensing by the door sensor, decreased steadily in both groups throughout the study.

Willingness to pay for the entire integrated home IoT system and services was higher in the prefrailty group compared with the frailty group throughout the study. The acceptable price was 7.04 dollars and 5.74 dollars in the prefrailty and frailty groups, respectively, at baseline. However, the price declined steadily to 3.07 dollars and 2.18 dollars, respectively, at 12 months (Supplementary Table 1).

### Health-related benefits and care burden

Health-related benefits of the services were reported with a 5-point scale, ranging from 1 (“strongly disagree”) to 5 (“strongly agree”). The scores on the items, “Feeling aided in performing physical functions” and “Relieving anxiety about health status,” were high for automatic gas circuit breaking (4.75 and 4.55, respectively) and remote light control (4.4 and 4.35, respectively) after participants had used all 8 services for 12 months. Perceived care burden of the participants was also reported with a 5-point scale, ranging from 1 (“very decreased”) to 5 (“very increased”). The occasion of making an urgent call to family was expected to be 1.85 (baseline) and decreased to 1.3 (final), and the occasion of needing acute hospital care was expected to be 1.9 (baseline) and increased to 2.8 (final).

### Changes with regard to the most satisfying service

**Figure 4:**
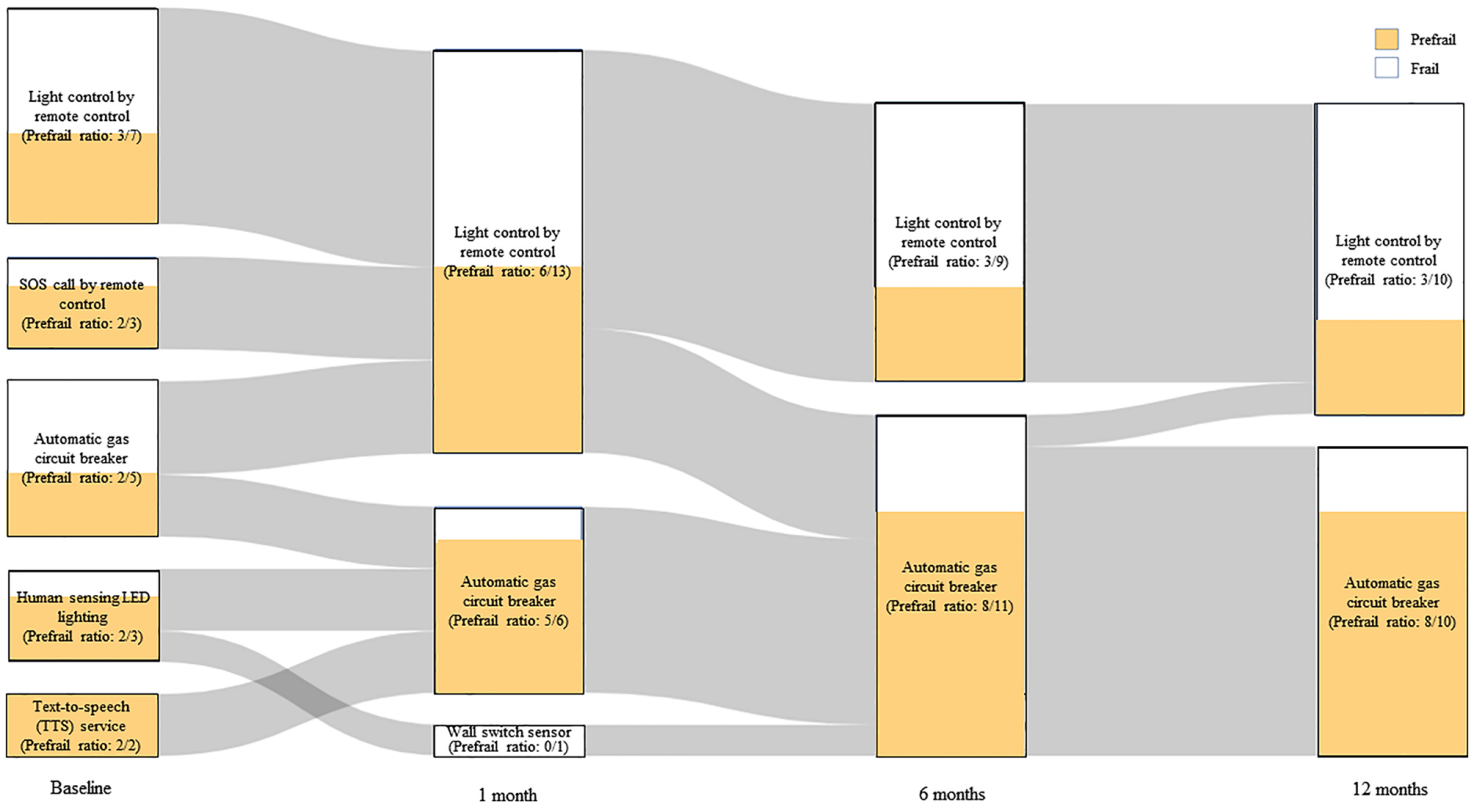
Changes with regard to most satisfactory service throughout the study. Unlike the baseline of the study, the most satisfactory service was narrowed to light control by remote controller and automatic gas circuit breaker for the frail and prefrail group, respectively. Data preprocessing was conducted by using Python software, version 3.6.8 (The Python Software Foundation, DE, USA), URL https://www.python.org, and the figure was created by using R software, version 4.1.0 (R Foundation, Vienna, Austria), URL https://www.r-project.org.

At baseline, there was considerable variety in the responses to the question regarding the most satisfying service (Fig. 4). However, as the study progressed two services gradually became the most satisfying among the participants: remote light control and automatic gas circuit breaking.

In the frailty group, remote light control, automatic gas circuit breaking, SOS call by remote control, and motion sensing by the sensor on the entrance ceiling lighting were expected to be the most satisfying at baseline, in that order. As the study progressed, remote light control and automatic gas circuit breaking gradually became the most satisfying services. By 12 months, remote light control was considered the most satisfying service in the frailty group (77.8%). In the prefrailty group, the initial satisfaction was almost evenly distributed among five services: remote light control, SOS call by remote control, automatic gas circuit breaking, motion sensing by the sensor on the entrance ceiling lighting, and TTS service. Similar to the frailty group, as the study progressed, remote light control and automatic gas circuit breaking gradually became the most satisfying services. By 12 months, gas circuit breaking was considered the most satisfying service in the prefrailty group (72.7%).

## Discussion

After the 12-month application of integrated home IoT services, involving five devices with eight different functions, in vulnerable older adults who were expected to have a high necessity of home IoT services, overall usability of the home IoT services was higher in prefrail older adults not in frail older adults. Further, the most satisfactory service of the participants differed according to their frailty status. We also found that the type of services determined usability pattern and unawareness of home IoT services existed both in prefrail and frail older adults. Vulnerable older adults did not recognize the real usefulness of IoT services before they practically used them, suggesting a potential digital literacy.

In previous studies, home IoT services and smart homes were found to benefit older adults by making it easier to manage their health, through such features as monitoring of health status. Fall detection and emergency notifications to health care providers through home IoT technologies made older adults raise their confidence and sense of security^34^. Sleep disturbance, anxiety, depression, and cognitive decline were attenuated owing to smart home system in community-dwelling older adults with dementia^7,35^. Also, video screen technology at home facilitated communication and improved quality of life of older adults^36^. In line with these findings, we also found that home IoT services for assisted vulnerable older adults in performing physical function, relieving anxiety, and reducing care burden. However, vulnerable older adults have not fully benefited from available home IoT services although they have the highest need. Usability becomes a staple factor in the adoption of digital technologies, as potential users may have difficulties in using devices due to their health condition and lack of experience^37^. In consequence, digital industries have devised numerous methods to measure perceived usability simply and reliably, mainly through questionnaires including the SUS^38^. Likewise, the usability issue is now regarded as a major barrier for the acceptance of home IoT systems and wearable devices especially for older adults^9,39^. In a study investigating the link between compliance of an activity tracker and health outcomes, the overall adherence rate was only 10%, and 40% of participants abandoned the device within 6 months^40^. Older adult perceptions regarding new technology are often negative. They are likely to perceive new technology as complex or as an invasion of privacy, be unaware that the technology exists, or find the device unreliable, which causes low usability^9,10^. In practice, the narrative review reported that easy manual of use and interaction with the devices were essential for the acceptance of smart homes especially in older adults^41,42^. Also, user-friendly interface and effective communication system were emphasized for better adoption of IoT systems in geriatric population^39^.

Usability was relatively high throughout the present study compared with previous studies, even though our participants were vulnerable older adults who lived without a caregiver, smart phone, or communication infrastructure at home. This may be because we constructed a home IoT system based on the demands and needs of older adults, which was examined in our previous study^21^. Particularly, the usability of IoT services considerably differed in accordance with the service itself and frailty status. There were certain services that showed high usability in all participants, whereas for some services, usability was dependent on frailty status. Of note, older adults with prefrailty showed higher usability in both total and individual services compared with older adults with frailty, which was in line with our previous report that compliance of wearable devices was higher in the prefrailty group (36.4%), not in the robust group (18.2%), and health outcomes significantly improved only in the prefrailty group^31^. From the perspective of geriatric care, frailty is a comprehensive concept representing overall health status. Unlike older adults with frailty who always need assistance and support, older adults with prefrailty can live independently with little assistance because their disability and decline in physical function are mild. Therefore, older adults with prefrailty may have more interests in healthcare devices and services than those with frailty and the frailty-centered approach in selecting subjects and services may offer an alternative solution of widening the scope and improving the usability of home IoT services for vulnerable older adults.

Another important finding of the study is that there was a perception change with regard to the most satisfying service before and after using the service. Early in the study, participants indicated being satisfied with five items; however, two services (remote light control and automatic gas circuit breaking) were preferred at the end of the study (Fig. 4). This change may be due to lack of awareness of IoT services or low digital literacy. In our previous face-to-face survey, only 1 percent of vulnerable older adults were currently using IoT technology and 12.5 percent had heard of IoT devices compared with 20 percent and 66 percent, respectively in vulnerable young adults^21^**.** The same study also indicated that there was a discordance in perspectives regarding service needs between healthcare providers and older adults^21^. Participants’ views of the most satisfactory services changed throughout the present study though we provided a service package that was expected to best meet the needs of older adults. At the end of the study, the most satisfying service was identical with the one which healthcare providers expected to be needed in our previous study^21^. This might suggest that older adults did not fully understand IoT services before they actually used those services, which means education for decreasing the perception gap is imperative. Given that older adults have a willingness to use IoT technology^21^, it is important to provide education and experience to help increase understanding of each service in advance, and giving frequent opportunity to interact with other users and service providers may be the key to crossing the barriers of usability and facilitating wider implementation.

Our study had several limitations. First, the study was conducted in a specific rural area of a single country. Since healthcare systems differ among countries due to differences in economic status, network infrastructure, and the services provided, our study results may not be generalizable to older adults in other countries. However, selected sample from ASPRA cohort might have representativeness of prefrail and frail older adults in Korean rural areas as we have already identified that ASPRA participants could represent the general Korean rural population^20^. In addition, given that we considered the specific characteristics of vulnerable older adults and carried out a comprehensive geriatric assessment, this frailty-based approach in selecting recipients and organizing services may be applicable to other countries and races. Furthermore, the system, which was designed for an environment of poor digital literacy and targeting vulnerable older adults, may be applicable in different situations. Second, the small sample size and high proportion of females may have introduced selection bias. However, from our consistent series of studies^19,21,43^, a long study period and high adherence rate with frequent face-to-face surveys on each service from a real-world academic-public health collaborative project would be beneficial for quickly incorporating this research result into policy and the market. Lastly, we did not systematically evaluate participants’ willingness to pay. However, this part was not a primary outcome of the study and may not be entirely accurate due to the following participant characteristics: impaired cognitive function, and mild disability, and difference in service costs by country. Therefore, it would be better to focus on relative change over time.

## Conclusions

When implementing home IoT services in vulnerable older adults with the highest need, the frailty-based approach is essential to maintain high usage and satisfaction. Also, providing tailored services based on frailty status and educating individuals to decrease digital literacy before and during the service, may lead to wider adoption of integrated home IoT services. Future research examining whether there are health benefits and changes in care burden compared with a control group, will be clinically meaningful for the application of integrated home IoT services as an effective home care service model for vulnerable older adults in the real-world setting.

## Supplementary Information

Supplementary Table 1.

## Data Availability

The datasets generated by and/or analyzed during the current study are not publicly available due to the policy of the AMC IRB, which does not allow the opening and sharing of research data with any third party, but they are available from the corresponding author upon reasonable request.
