# Supplementary material for: Implementation of an integrated home internet of things system for vulnerable older adults using a frailty-centered approach
Source: Sci Rep. 2022 Feb 4;12:1922. doi: 10.1038/s41598-022-05963-9 (PMC8817027; doi:10.1038/s41598-022-05963-9)
Supplement: Supplementary file 1 — Supplementary Table 1. [file 41598_2022_5963_MOESM1_ESM.docx]

Implementation of an integrated home Internet of Things system for socioeconomically vulnerable older adults using a frailty-centered approach

Ji Yeon Baek, MD, PhD^1^; Se Hee Na, R.Ph^2^, Heayon Lee MD,PhD^3^, Hee-Won Jung, MD, PhD^1^; Eunju Lee, MD, PhD^1^; Min-Woo Jo, MD, PhD^4^; Yu Rang Park, PhD^2*^, Il-Young Jang, MD^1*^

^1^Division of Geriatrics, Department of Internal Medicine, Asan Medical Center, University of Ulsan College of Medicine, Seoul, Republic of Korea

^2^Department of Biomedical System Informatics, Yonsei University College of Medicine, Seoul, Republic of Korea

^3^Division of Pulmonary, Critical Care and Sleep Medicine, Department of Internal Medicine, Eunpyeong St. Mary’s Hospital, The Catholic University of College of Medicine, Seoul, Republic of Korea

^4^Department of Preventive Medicine, University of Ulsan College of Medicine, Seoul, Republic of Korea

Address correspondence to:

**Yu Rang Park, PhD**

Department of Biomedical Systems Informatics, Yonsei University College of Medicine

50-1 Yonsei-ro Seodaemun-gu, Seoul 03722, Republic of Korea

Tel: +82-2-2228-2493; Fax: +82-2-2228-2493; E-mail: yurangpark@yuhs.ac

**Il-Young Jang, MD**

Division of Geriatrics, Department of Internal Medicine, Asan Medical Center, University of Ulsan College of Medicine

88 Olympic-ro 43-gil, Songpa-gu, Seoul 05505, Republic of Korea

Tel: +82-2-3010-1658; Fax: +82-504-476-9099; E-mail: onezero2@gmail.com

**Supplementary information**

**Supplementary Table 1** Willingness to pay for the services

| Timepoint | Prefrailty Group (n=11) | Frailty Group (n=9) | Total (n=20) | *p* value |
| --- | --- | --- | --- | --- |
| Baseline | 7.04 (2.96) | 5.74 (2.14) | 6.45 (2.64) | 0.047 |
| 1 month | 5.10 (2.79) | 4.01 (1.86) | 4.61 (2.42) | 0.172 |
| 6 months | 3.88 (2.12) | 3.26 (1.60) | 3.61 (1.88) | 0.359 |
| 12 months | 3.07 (1.84) | 2.18 (1.27) | 2.67 (1.63) | 0.122 |

Values are presented as mean±standard deviation and are based on the United States dollar.
